# Feasibility and acceptability of a novel telepsychiatry-delivered precision prescribing intervention for anxiety and depression

**DOI:** 10.1186/s12888-022-04113-9

**Published:** 2022-07-19

**Authors:** Erin O’Callaghan, Scott Sullivan, Carina Gupta, Heather G. Belanger, Mirène Winsberg

**Affiliations:** 1 Brightside Health Inc., 2471 Peralta Street, Oakland, CA 94607-1703 USA; 2 Departments of Psychology and Psychiatry and Behavioral Neurosciences, University of South Florida, Tampa, FL USA

**Keywords:** Telemedicine, Psychiatry, Mental health, Depression, Anxiety

## Abstract

**Background:**

Major Depressive Disorder and Generalized Anxiety Disorder are pervasive and debilitating conditions, though treatment is often inaccessible and based on trial-and-error prescribing methods. The present observational study seeks to describe the use of a proprietary precision prescribing algorithm piloted during routine clinical practice as part of Brightside’s telepsychiatry services. The primary aim is to determine the feasibility and acceptability of implementing this intervention. Secondary aims include exploring remission and symptom improvement rates.

**Methods:**

Participants were adult patients enrolled in Brightside who completed at least 12 weeks of treatment for depression and/or anxiety and received a prescription for at least one psychiatric medication. A prescription recommendation was made by Brightside’s algorithm at treatment onset and was utilized for clinical decision support. Participants received baseline screening surveys of the PHQ-9 and GAD-7, and at weeks 2,4,6,8,10 and 12. Intent-to-treat (ITT) sensitivity analyses were conducted. Feasibility of the implementation was measured by the platform’s ability to enroll and engage participants in timely psychiatric care, as well as offer high touch-point treatment options. Acceptability was measured by patient responses to a 5-star satisfaction rating.

**Results:**

Brightside accessed and treated 6248 patients from October 2018 to April 2021, treating a majority of patients within 4-days of enrollment. The average plan cost was $115/month. 89% of participants utilized Brightside’s core medication plan at a cost of $95/month. 13.4% of patients in the study rated Brightside’s services as highly satisfactory, averaging a 4.6-star rating. Furthermore, 90% of 6248 patients experienced a MCID in PHQ-9 or GAD-7 score. Remission rates were 75% (final PHQ-9 or GAD-7 score < 10) for the study sample and 59% for the ITT sample. 69.3% of Brightside patients were treated with the medication initially prescribed at intake.

**Conclusions:**

Results suggest that the present intervention may be feasible and acceptable within the assessed population. Exploratory analyses suggest that Brightside’s course of treatment, guided by precision recommendations, improved patients’ symptoms of anxiety and depression.

## Background

Major Depressive Disorder (MDD) is one of the leading causes of disability in the United States that affects more than 16.1 million adults each year [1], though only 65% of people suffering from depression receive treatment [2]. Often co-occurring with depression, Generalized Anxiety Disorder (GAD) is also a pervasive and debilitating condition affecting 6.8 million adults nationally, yet only about 43% of individuals receive treatment [1]. While a significant number of adults suffer from mental health problems like anxiety and depression in the United States, only some receive adequate care, with reported barriers to seeking treatment contributing to an unmet need for accessible, quality care options. Key barriers to receiving care that can account for this treatment gap include lack of health insurance and financial resources, limited availability of providers, transportation difficulties, stigma, cultural competencies, and distress associated with having a psychological impairment [3–5]. Importantly, many of these barriers involve structural difficulties to accessing mental health services, such as lack of safe infrastructure and commute options, shortage of hospitals and licensed specialty providers [6, 7]. Research has shown that specialty care tends to be concentrated to urban, populated areas, often isolating rural communities from evidence-based treatment options and rendering them out of reach [6, 7]. This disparity is highlighted by findings that while the gold standard of psychiatric treatment for mental health disorders includes measurement-based psychiatric care with regular follow-ups, optimizing ongoing treatment decisions based on individualized outcomes [8], the majority of people with anxiety and/or depression in the United States are treated in an unspecialized primary care setting with medication management [9]. Many primary care providers in rural treatment settings report a significant unmet need for increased access to evidence-based treatment modalities and specialized psychiatry services [10].

Over the last two decades, a rich literature has evolved outlining the promise of digital mental health care options like telepsychiatry that can help eliminate structural barriers to evidence-based care [11–21].Telepsychiatry refers to the use of electronic communication to provide psychiatric care at a distance, rather than through an in-person meeting between patient and provider [11]. Research has shown that the innovation of telehealth has helped to eliminate physical and geographical barriers to evidence-based treatment, even offering a centralized, digital workspace for psychiatric providers to collaborate across great distances [22–24]. Studies also suggest that telepsychiatry treatments for anxiety [16] and depression [14] may be beneficial, offering lower attrition rates than those observed in traditional in-person care. These data underscore telepsychiatry as an accessible and effective treatment delivery option with the potential to increase widespread availability of evidence-based mental health care.

Particularly during the COVID-19 pandemic, a period marked by unprecedented structural barriers to care across patient demographic lines, telepsychiatry services have seen extraordinary popularity and growth, with governments and organizations across the globe encouraging the use of telepsychiatry services to offer imminent and continued access to services [25]. As providers closed their brick-and-mortar locations in an attempt to reduce infection risk, many were forced to realize the full potential of digital tools amid rising demand from individuals affected by anxiety, grief, fear of contamination, isolative depression during quarantine, and socioeconomic impacts that have marked life during the COVID-19 pandemic [26]. Accordingly, leveraging this surge in acceptance and utility of digital health platforms has largely been seen as an opportunity for the field to expand access to evidence-based mental health care [26].

As mental health platforms and applications have become more prevalent, multiple studies have been conducted to assess their efficacy in the treatment of psychiatric illness, particularly depression and anxiety disorders. Meta-analysis of these studies has demonstrated that patients using mobile applications showed greater improvement in symptoms than controls, with the effect greater when compared to inactive control conditions [27, 28]. Many of these mobile applications empower patients by providing them with the ability to track their progress, thus become an active participant in their treatment. Applications may also serve as useful tools for symptom monitoring, thereby facilitating early identification of risk, and mitigating negative psychiatric outcomes [29].

For those who do have access to and receive evidence-based psychiatric care, several factors highlight the need for a more precise means of treatment selection that expands on current standards for patients with anxiety and depression. At present, only about 50% of psychiatric drug treatment choices are successful [30]. Unlike most medical fields to date, modern psychiatric practice largely remains based on subjective symptoms and observations charted throughout the patient-provider relationship [31, 32]. As such, traditional symptom-only based categories, like those constructed in the DSM-5 and ICD-10 manuals, exclude biological validity, lending such models to a level of heterogeneity [31, 32]. There exists a critical need for personalization of treatment within psychiatry, by means of selecting treatments that are effective and avoiding those that may not serve a particular patient.

Precision medicine has shown promise as a method for optimizing treatment selection to improve outcomes and manage complex disease states. Precision medicine relies on “treatments targeted to the needs of individual patients on the basis of genetic, biomarker, phenotypic, or psychosocial characteristics that distinguish a given patient from other patients with similar clinical presentations.” [33] Recent advances in precision medicine have significantly changed diagnosis and treatment in areas such as oncology with clinicians now being able to take into account patients’ individual clinical and biological characteristics to inform clinical decision making [34]. The use of precision medicine in psychiatry is in its early stages compared to other medical fields [35], but there is promising early evidence for a paradigm shift toward more individualized diagnosis. For example, in the Sequenced Treatment Alternatives to Relieve Depression (STAR*D) study, it was found that risk for treatment-resistance among those with major depressive disorder can be predicted using various demographic and patient-reported clinical features (e.g., insomnia, psychosis, etc) [36].

In particular, research from the U.S. National Institute of Mental Health’s “precision medicine for psychiatry” project suggests that building a more precise psychiatric framework beyond current diagnostic categories can predict key outcome measures [37]. Utilizing Research Domain Criteria (RDoC), researchers have deconstructed diagnostic groups into biologically meaningful subgroups of mood disorders that have biological validity, though importantly, these subgroups do not map neatly onto existing psychiatric symptom clusters [38]. These insights suggest that empirically clustering patient symptoms and biological information beyond traditional models has potential for clinical utility.

More recently, the field of precision psychiatry has begun exploring the potential of advanced analytic methods, such as machine learning, to predict clinically useful treatment determinations [38]. Machine learning refers to the use of algorithmic methods to identify general data principles underlying a set of analytical observations without specific instructions, characterized by mining knowledge from Big data and limited formal assumptions, allowing the data to be self-explanatory [38]. Using data derived from the STAR*D study, machine learning algorithms were assessed alongside logistical regression methods to determine the automated potential of a variety of clinical variables to predict treatment resistance to antidepressants [36]. Both methods showed comparable ability to predict treatment resistance to depression across datasets [36]. Specifically, a logistic regression model achieved an area under the receiver operating characteristic curve (AUC) of 0.72 across cohorts, using demographic and patient-reported clinical factors (like insomnia, psychosis, etc), thereby providing clinicians with a useful clinical-decision support tool.

Many such algorithms have been incorporated into decision support (CDS) tools to assist providers in treating depression in making choices about the best clinical approach, with demonstrated efficacy in depression outcomes in primary care and general practices [39–41]. For example, the Texas Medication Algorithm Project (TMAP) [42] was developed as a CDS to incorporate treatment guidelines, based on the empirical literature and expert opinion, for use in primary care. While initially in paper and pencil format, a computerized decision support system was developed for TMAP and found to be superior to usual care [40].

Given the promise of telemental health care delivery, decision-support, and precision psychiatry methods for providing accessible, quality treatment of depression and anxiety, Brightside Health Inc. developed a proprietary precision prescribing algorithm, leveraging digital clinical decision support based on patient symptom clusters and existing research literature. The use of this algorithm has been piloted via telepsychiatry for large-scale clinical care across the United States. The primary goal of this study was to determine the feasibility and acceptability of implementing this algorithm in a virtual clinical setting. We also sought to calculate remission and symptom improvement rates. We hypothesized that the Brightside intervention would be feasible and acceptable within the defined population.

## Methods

### Participants

**Fig. 1:**
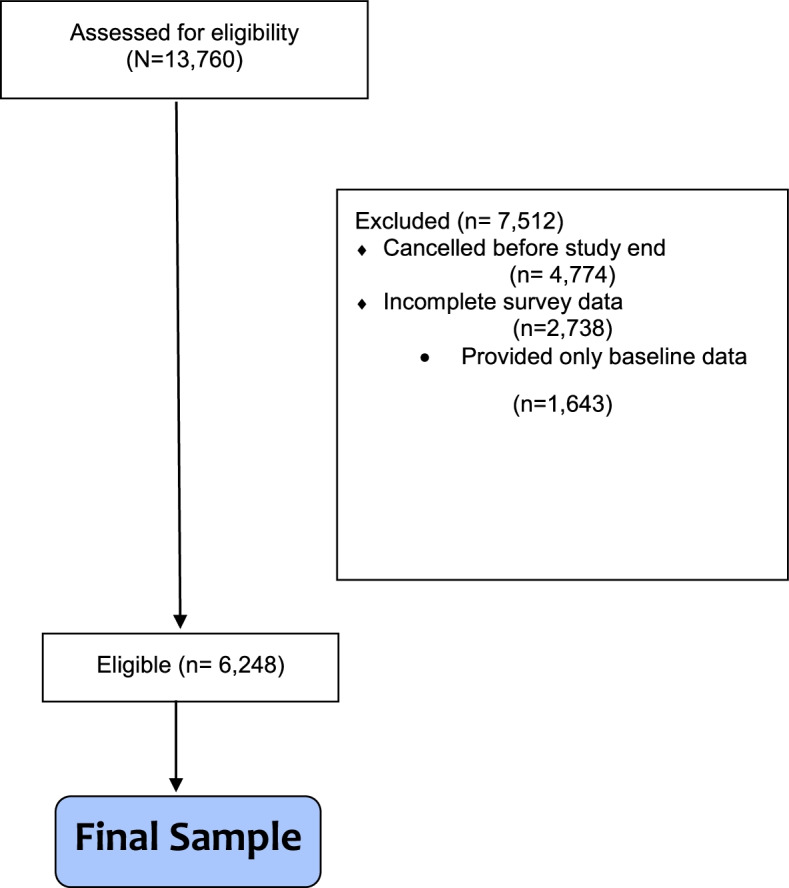
Eligible and ineligible participants

The Brightside dataset was constructed from the total population of patients receiving psychiatric care for depression and/or anxiety from Brightside during the period of October 2018 through April 2021. This included only Brightside members receiving psychiatric (and not psychotherapy) services, with a primary diagnosis of depression and/or anxiety, and a positive screen (10+) for depression or anxiety as measured by the Patient Health Questionnaire-9 (PHQ-9) or the Generalized Anxiety Disorder (GAD-7) questionnaire upon intake. Patients were included in the study if they had a minimum of 12 weeks of survey data and received a prescription for at least one psychiatric medication during that time. Patients were excluded if they had psychosis, schizophrenia, or bipolar 1 disorder, or chronic health conditions that would require active lab monitoring (e.g., chronic liver or kidney disease). The number of participants assessed for eligibility and the final sample are presented in Fig. 1. Intent-to-treat (ITT) sensitivity analyses were completed, which included participants excluded because they either cancelled their Brightside treatment plan before the completion of 12 weeks, or they provided incomplete data (i.e., less than 12 weeks).

### Procedure

The WCG Institutional Review Board approved this retrospective protocol. Brightside’s telemental health platform offers psychiatric services via a web-based interface, using a measurement-based approach to track outcomes and provide clinical decision support to clinical providers. The Brightside platform provides this decision support via a computerized symptom cluster analysis (see symptoms clusters in Table 5) at treatment intake. Based on analysis of presenting symptom clusters, as well as decision support based on the empirical literature, treatment recommendations are provided. After treatment begins, patients are monitored via the platform using the PHQ-9, GAD-7 and other proprietary questions to track progress and surface concerns to the treating provider. Providers are alerted in real time when patients fail to improve, worsen, or experience symptoms such as suicidal ideation. Brightside providers, most of whom were primary care physicians at the time of this study, communicate with their patients both asynchronously via electronic messaging and synchronously via live video sessions. Precision prescribing, coupled with measurement-based follow-up, all delivered via telehealth, differentiates Brightside care.

Brightside clinicians prescribe from a wide array of psychotropic medications. Treatment recommendations can include over 300 different prescription combinations (see Table 1 for the initial prescription categories of the study sample). The focus of this study is on Brightside’s psychiatric and medication services, though Brightside also offers psychothearapy.

**Table 1:** Initial prescription medication classes of study sample

Drug Class	Study Sample	
	(*N* = 6248)	
SSRI Only	3583 (57.3%)	
NDRI Only	1202 (19.2%)	
SNRI Only	331 (5.3%)	
Other	256 (4.1)	
Combination:		
Including SSRI	674 (10.8%)	
Including NDRI	132 (2.1%)	
Including SNRI	70 (1.1%)	

*SSRI* Selective serotonin reuptake inhibitor, *NDRI* Norepinephrine and dopamine reuptake inhibitor, *SNRI* Serotonin and norepinephrine reuptake inhibitors, Other = mirtazapine, tricyclics, trazodone, second generation antipsychotics, gabapentin, anticonvulsants, alpha agonists, beta blockers, and/or combinations of these. Combinations include at least more than one medication

Surveys of the PHQ-9 and GAD-7 were administered digitally through an email prompt at baseline before the start of treatment, and then at 2,4,6,8,10, and 12 weeks. Clinical touchpoints included synchronous video consults, asynchronous provider messages sent and received, case-reviews and completed surveys. Survey completion at start and endline of study were required for participation. Endline surveys were collected at week 12, or within a 4-week buffer period after week 12 if the participant did not submit one exactly at week 12. At 4–8 weeks after initiation of treatment, participants were prompted by Brightside to rate their satisfaction with the platform’s services.

### Measures

The feasibility of implementing Brightside’s precision prescribing algorithm via telepsychiatry as part of routine clinical practice was measured by the platform’s ability to enroll and engage participants in timely, cost-effective psychiatric care, as well as offer dynamic, high touch-point treatment options throughout the duration of the study. Acceptability of Brightside’s methodology was examined by patient responses to a 5-star, Likert satisfaction scale with the prompt, “how satisfied were you with Brightside’s services?” One star represented unsatisfied and five stars represented very satisfied. The PHQ-9 is a 9-question self-report measure of depressive symptom severity. Respondents rate each item on a four-point Likert scale (0–3) with total scores ranging from 0 to 27 (higher scores reflect greater depression severity). The PHQ-9 demonstrates strong reliability and validity with 88% sensitivity and 88% specificity for major depressive disorder (MDD) [43]. It is sensitive to antidepressant response [44].

The GAD-7 is a 7-item self-report measure of Generalized Anxiety Disorder (GAD) symptoms with a four-point Likert scale and a total score ranging from 0 to 21. Like the PHQ-9, a higher score corresponds to a greater anxiety severity. The GAD-7 has good psychometric properties with 89% sensitivity and 82% specificity for GAD [45, 46].

Based on response to intake questions, Brightside patients are categorized by symptom clusters, and one or more symptom clusters are surfaced to the clinician. These include severe melancholic depression, presence of anxious distress, atypical features of depression, prominent anxiety, insomnia, presence of chronic pain, cardiovascular risk factors, pregnancy or breastfeeding, or prior success with a medication trial.

Data analyses were performed using SPSS to assemble the patient data sample, apply inclusion and exclusion criteria and establish baseline versus endline survey outcomes. Brightside maintains de-identified SQL databases for analytics that facilitate granular insights into clinical decisions, interactions and outcomes. Specifically, the key outcomes computed included the proportion of patients experiencing a minimal clinically important difference (MCID) in PHQ-9 (5+ point reduction) [47] and/or GAD-7 (4+ point reduction) [48] scores and the proportion of patients achieving remission with a < 10 score on PHQ-9 and/or GAD-7 after starting with corresponding baseline score of 10 or greater.

## Results

A total of 6248 patients were enrolled in Brightside from October 2018 to April 2021 and completed at least 12-weeks of treatment with Brightside (Table 2). As can be seen in Table 2, there were statistically significant, but negligible differences between the study sample and those who were ineligible. The study sample contained 5% more white participants. Patients averaged 3.7 days from the time of enrollment to first appointment (50.8% of patients were treated within 48 hours of enrollment, 68.7% within 72 hours, 78.9% within 96 hours.) Study patients experienced an average of 15.5 clinical touchpoints throughout the 12-week study period, which included synchronous video consults, asynchronous provider messages sent and received, case-reviews and check-in surveys. 13.4% of patients in this study submitted a review of their experience with Brightside’s services, with an average rating of 4.6 stars. Of those that submitted a review, 72.5% rated their experience with 5-stars.

**Table 2:** Demographic characteristics of study and ineligible samples

Characteristic	Study Sample	Ineligible		
	(*n* = 6248)	(*n* = 7512)	*p-*value	*V*
Sex			.04	.02
Female	4452 (71%)	5224 (70%)		
Male	1796 (29%)	2286 (30%)		
Age			<.001	.06
18–24	791 (13%)	1249 (17%)		
25–34	3424 (55%)	3876 (52%)		
35–44	1451 (23%)	1742 (23%)		
45–54	433 (7%)	473 (6%)		
55–64	131 (2%)	137 (2%)		
> 64	18 (< 1%)	35 (< 1%)		
Ethnicity			<.001	.07
White/European	4994 (80%)	5631 (75%)		
Hispanic/Latino	474 (8%)	717 (10%)		
Other/Mixed	313 (5%)	425 (6%)		
Asian	210 (3%)	330 (4%)		
Black/African American	203 (3%)	358 (5%)		
Native American	30 (< 1%)	35 (< 1%)		
Pacific Islander	24 (< 1%)	16 (< 1%)		
Education			<.001	.07
< High School	81 (2%)	155 (2%)		
High School	1821 (29%)	2495 (33%)		
Associate’s degree	878 (14%)	1217 (16%)		
Bachelor’s degree	2398 (38%)	2491 (33%)		
Advanced degree	1070 (17%)	1154 (16%)		
Annual Income			<.001	.05
< $30 K	1956 (31%)	2713 (36%)		
$30 K-$60 K	2035 (33%)	2372 (32%)		
$60 K-$100 K	1304 (21%)	1365 (18%)		
> $100 K	953 (15%)	1062 (14%)		
Geographic Region			.18	.01
South	2007 (32%)	2538 (34%)		
West	1764 (28%)	2092 (28%)		
Midwest	1261 (20%)	1442 (19%)		
Northeast	1216 (20%)	1440 (19%)		

*V* = Cramer’s *V* (values <.1 considered a negligible effect size [49])

Study patients skewed female, with 71% identifying as female, and saw the greatest proportion of patients in the 25–34-year-old age bracket with a mean age of 31.6 years (Table 2). Approximately 80% of study patients identified as White (Table 2). Income levels of patients varied; 31% of the patients earned less than $30,000 income per year (Table 2).

**Fig. 2:**
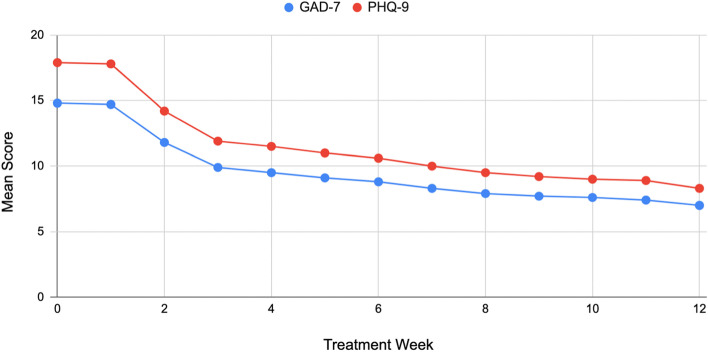
Mean PHQ-9 and GAD-7 scores over 12-week study period

Mean PHQ-9 score at baseline was 17.9. PHQ-9 and GAD-7 scores are presented in Table 3. Mean PHQ-9 and GAD-7 scores across all 12 weeks are presented in Fig. 2. The depressive severity seen in study participants as measured by baseline PHQ-9 was distributed as 4% mild, 20% moderate, 37% moderate-severe, and 39% severe. The ineligible group had similar scores at baseline (Table 3), but there was significantly greater change in the study sample, and significantly lower scores at 12 weeks, both with medium effect sizes [50].

**Table 3:** Survey means (standard deviation) by group

Survey	Study Sample	Ineligible		
	(*n* = 6248)	(*n* = 7512)	*p-*value	*d*
PHQ-9				
Baseline	17.90 (4.73)	18.15 (4.96)	.002	.05
Endline	8.29 (5.59)	12.56 (6.38)	.001	.67
Change	−9.61 (5.97)	−5.59 (5.93)	<.001	.64
GAD-7				
Baseline	14.79 (4.50)	15.27 (4.50)	<.001	.11
Endline	7.00 (5.25)	10.83 (6.03)	.001	.64
Change	−7.79 (5.82)	−4.44 (5.53)	<.001	.57

For ineligible participants, the “Endline” score is the last score provided by that individual. *d* = Cohen’s *d* (a *d* of .2 is considered a small effect size, .5 a medium effect, and .8 a large effect) [50]

90% of study patients experienced a minimally clinically important reduction (MCID) from baseline to 12 weeks (Table 3), as compared to 61% of those who were ineligible. 81% of study patients experienced a minimally clinically important reduction (MCID) on the PHQ-9 from baseline to 12 weeks (Table 3). Of the patients achieving remission at 12 weeks, 65% of patients achieved remission by 28 days, and 82% by 48 days (Table 4). Mean time to remission was 31 days. Remission rates were 75% as measured by a final PHQ-9 or GAD-7 score below 10, and remission rates were 63% as measured by a final PHQ-9 score below 10 (Table 4). There were significant differences in MCID and remission rates between the study sample and those who were ineligible. Symptom clusters associated with highest rates of remission were anxiety, insomnia and core emotional features of depression (Table 5). Symptom clusters associated with lower rates of remission were severe melancholic subtypes, presence of chronic pain, anxious distress subtypes, and atypical features (Table 5).

**Table 4:** Frequency of MCID and remission outcomes at endline

Outcome Type	Survey Basis	Eligible No. Sample/Ineligible	Reporting Outcome No. (%) Study Sample (***n*** = 6248)	Reporting Outcome No. (%) Ineligible (***n*** = 7512)	Reporting Outcome No. (%) Intent-to-Treat Sample (***n*** = 13,760)	***p***-value	***V***
MCID	PHQ-9 and/or GAD-7	6248/7512	5599 (90%)	4585 (61%)	10,184 (74%)	<.001	.32
MCID	PHQ-9	6248/7512	5031 (81%)	3911 (52%)	8942 (65%)	<.001	.30
MCID	GAD-7	6248/7512	4760 (76%)	3622 (48%)	8382 (61%)	<.001	.30
Remission	PHQ-9 and/or GAD-7	6248/7512	4716 (75%)	3341 (44%)	8057 (59%)	<.001	.31
Remission	PHQ-9	6005/7149	3785 (63%)	2385 (33%)	6170 (47%)	<.001	.30
Remission	GAD-7	5368/6634	3714 (69%)	2588 (39%)	6302 (53%)	<.001	.30

*P* and *V* values are for chi-square comparison between study sample and ineligible sample. *V* = Cramer’s *V* (values between .2 and .4 are considered a moderate effect size [49])

**Table 5:** Symptom cluster overview

Symptom Cluster / Clinical Decision Support Segment	% of Patient Count	Patient Count
Atypical Features	24.4%	1526
Previous Prescription Success	23.1%	1440
Anxiety	15.4%	964
Insomnia	14.0%	877
Hypertension / Hyperlipidemia	7.0%	435
Severe Melancholic	5.8%	361
Chronic Pain/ Headaches/ Fibromyalgia	5.0%	313
Anxious Distress	1.7%	109
Core Emotional	1.7%	105
Cardiac Arrhythmias	0.7%	41
Risk of Pregnancy/Breastfeeding	0.5%	30
Generalized	0.5%	31
Coronary Artery Disease	0.3%	16
**Grand Total**	100.0%	6248

Brightside study patients in the study received over 1000 medication/dose combinations, and more than half of patients had at least 1 medication adjustment within the study period. Using a symptom cluster approach and data driven clinical decision support, 69.3% of Brightside study patients were treated with the medication initially prescribed at intake (irrespective of medication dosage), whereas 30.7% required further iteration of their treatment plan with an augmentation strategy or switch.

## Discussion

Telepsychiatry has shown promise as an emerging solution for inhibited access to quality psychiatric care in the United States. Additionally, leveraging precision prescribing to deliver large-scale precision psychiatry insights has demonstrated progress as a means of advancing traditional and often heterogeneous psychiatric prescribing methods.

The present analysis sought to investigate the feasibility and acceptability of a proprietary precision prescribing algorithm via telepsychiatry for large-scale, routine clinical care for anxiety and depression across the United States. We also sought to explore the potential effectiveness of this algorithm to identify appropriate courses of treatment at onset and subsequently assess associated symptom improvement and remission rates.

Ultimately, Brightside was able to treat 6248 patients from October 2018 to April 2021, a period that included an unprecedented period of structural access to psychiatric care as a result of the COVID-19 pandemic. There were not appreciable differences between those who completed 12 weeks of treatment and those who did not. The platform was able to provide timely psychiatric care, treating a majority of patients within 4-days of enrollment, as compared to evidence gathered across three major cities in the United States that suggests average wait times for psychiatry appointments can be an average of 25 days [51]. Furthermore, 89% of participants paid $95 per month for psychiatric services as compared to a higher average cost for an initial psychiatric evaluation plus follow-up. This rate compares favorably to Ruskin et al.’s [52] estimated marginal costs of $86.16 for a single telepsychiatry session. The estimated cost of one month of in-person psychiatry services is about $600 [53]. The cost of telepsychiatry is widely debated and discussed with various ways of calculating potential cost savings [54, 55].

Of the Brightside study sample, 90% of study patients (and 74% of the ITT sample) experienced a minimally clinically important reduction (MCID) from baseline to 12 weeks. The mean change of − 9.6 on the PHQ-9 compares favorably to a mean change of − 7.5 in the Improving Mood-Promoting Access to Collaborative Care (IMPACT) cohort over the same time period [47]. Remission rates were 75% as measured by a final PHQ-9 or GAD-7 score below 10, and 59% in the overall ITT sample. These rates compare favorably to a remission rate of 28% in the initial 14 weeks of treatment in the STAR*D trial with generally milder depression [56], though a different outcome measure was used in that study. Clinical trials for depression report remission rates of 22 to 40%, while studies including representative samples of depressed patients more similar to those seen in actual clinical practice report lower remission rates of 11 to 30% [57]. Clearly, the Brightside study sample, whether inclusive of those who dropped out or not, fared well relative to these historical numbers. There were significant differences in MCID and remission rates between the study sample and those who were ineligible, with medium effect sizes. As the ineligible group included a large portion (64%) who received at least some treatment, these effect sizes are likely an underestimate of the true treatment effect.

Remission rate is particularly important because those who achieve remission with treatment will likely have higher functioning [58] and improved prognosis [59], relative to non-remitters, over time, though further follow-up will be needed to assess whether the remission is sustained in the Brightside patients.

Though only 13% of patients provided satisfaction data, the average rating was 4.6 stars, with 72.5% rating their experience with 5-stars. Ruskin et al. [52] similarly reported overall good satisfaction (i.e., ‘agree’ to ‘strongly agree’) with their telepsychiatry platform. In general, patients report high satisfaction with telepsychiatry services [22, 60], with particularly high ratings for financial and accessibility/convenience [61]. A recent review of depression trials found that satisfaction with telepsychiatry was equivalent to or significantly higher than to face-to-face [62]. Our low response rate overall, however, with only 15% of participants responding, tempers our ability to draw conclusions regarding satisfaction with the Brightside platform.

Results of the current study should be considered in light of its design limitations. Importantly, the current investigation was not a clinical trial. As such, conclusions cannot be drawn regarding the effectiveness of Brightside’s precision prescribing method as a stand-alone treatment method for reducing symptoms of anxiety and depression. Along the same vein, this study lacks a control group to compare the outcomes of Brightside patients to outcomes of patients treated with standard of care. Furthermore, while patients receiving psychotherapy services through Brightside were excluded to prevent introducing a confounding variable, it is unknown if patients were receiving psychotherapy independent of the platform.

The present study also contains key biases, such as self-selection bias, as patients independently seek out care. Survivorship bias. This sample also biases results toward a majority White female population, revealing challenges recruiting a sample with wider race and sex diversity, although socioeconomic status showed notable variance. Lastly, information about marital status was unknown, so it was not possible to determine whether married participants fared better in our sample, as research has previously shown [63]. These biases limit the generalizability of the present study’s findings.

This study also utilized a patient-reported tool, the PHQ-9, to measure patient symptoms of depression over the course of treatment and did not utilize a clinician-reported measurement tool such as the Hamilton Depression Rating Scale (HDRS)  [64]. It is important to note that the PHQ-9 is a brief measure of depression symptoms that is commonly used in clinical practice. While the HDRS is the measure typically used in clinical trials, its length is prohibitive in real-world clinical settings. Further, research has shown that patients’ reports of their own symptoms are generally very similar to clinician-reported measures of symptoms [65].

## Conclusions

These results suggest that Brightside’s intervention may be feasible within the defined population. Brightside was also well received among users. When given the opportunity to rate their experience with Brightside, a majority of patients submitted a 5-star review. Furthermore, as most patients were treated using the medication initially prescribed by Brightside’s prescribing algorithm, preliminary findings suggest that Brightside’s course of treatment may have improved patients’ symptoms of anxiety and depression.

Future study should utilize a randomized controlled trial or wait-list control group to assess the effectiveness of Brightside’s precision prescribing method, including strong measures of baseline sociodemographic and clinical characteristics, exploring potential mediators of treatment outcome.

## Data Availability

The datasets analyzed for the current study are available from the corresponding author on reasonable request.

## Abbreviations

DSM-5: Diagnostic and Statistical Manual of Mental Disorders, Edition 5
GAD-7: Generalized Anxiety Disorder Scale, 7-item instrument
GBDT: Gradient Boosting Decision Tree
HRSD: Hamilton Rating Scale for Depression
ICD-10: International Classification of Diseases, Edition 10
MCID: Minimally Clinically Important Differences
MDD: Major Depressive Disorder
PHQ-9: Patient Health Questionnaire, 9-item instrument
RDoC: Research Domain Criteria
STAR*D: Sequenced Treatment Alternatives to Relieve Depression Study
SQL: Structured Query Language
